# Air pollution exposure is associated with MRSA acquisition in young U.S. children with cystic fibrosis

**DOI:** 10.1186/s12890-017-0449-8

**Published:** 2017-07-27

**Authors:** Kevin J. Psoter, Anneclaire J. De Roos, Jon Wakefield, Jonathan D. Mayer, Margaret Rosenfeld

**Affiliations:** 1 Department of Pediatrics, School of Medicine, The Johns Hopkins University Bayview Medical Center, 5200 Eastern Ave, Mason F. Lord Bldg, Center Towers, Suite 4200, Baltimore, MD 21224 USA; 2 Department of Environmental and Occupational Health, Drexel University School of Public Health, Philadelphia, PA USA; 3 Departments of Biostatistics and Statistics, University of Washington, Seattle, WA USA; 4 Departments of Epidemiology, Geography, Global Health, Medicine (Allergy and Infectious Diseases), Family Medicine, and Health Services, University of Washington, Seattle, WA USA; 5 Division of Pulmonary Medicine, University of Washington School of Medicine, Seattle, WA USA

**Keywords:** Cystic fibrosis, *Staphylococcus aureus*, MRSA, *Stenotrophomonas maltophilia*, *Achromobacter xylosoxidans*, Fine particulate matter, Air pollution

## Abstract

**Background:**

The role of air pollution in increasing susceptibility to respiratory tract infections in the cystic fibrosis (CF) population has not been well described. We recently demonstrated that chronic PM_2.5_ exposure is associated with an increased risk of initial *Pseudomonas aeruginosa* acquisition in young children with CF. The purpose of this study was to determine whether PM_2.5_ exposure is a risk factor for acquisition of other respiratory pathogens in young children with CF.

**Methods:**

We conducted a retrospective study of initial acquisition of methicillin susceptible and methicillin resistant *Staphylococcus aureus* (MSSA and MRSA), *Stenotrophomonas maltophilia* and *Achromobacter xylosoxidans* in U.S. children <6 years of age with CF using the CF Foundation Patient Registry, 2003–2009. Multivariable Weibull regression with interval-censored outcomes was used to evaluate the association of PM_2.5_ concentration in the year prior to birth and risk of acquisition of each organism.

**Results:**

During follow-up 63%, 17%, 24%, and 5% of children acquired MSSA, MRSA, *S. maltophilia*, and *A. xylosoxidans*, respectively. A 10 μg/m^3^ increase in PM_2.5_ exposure was associated with a 68% increased risk of MRSA acquisition (Hazard Ratio: 1.68; 95% Confidence Interval: 1.24, 2.27). PM_2.5_ was not associated with acquisition of other respiratory pathogens.

**Conclusions:**

Fine particulate matter is an independent risk factor for initial MRSA acquisition in young children with CF. These results support the increasing evidence that air pollution contributes to pulmonary morbidities in the CF community.

## Background

Cystic fibrosis (CF) lung disease is characterized by a vicious cycle of chronic pulmonary inflammation and endobronchial infection. This process can begin early in life, as inflammation and structural airway damage are often present during infancy and the preschool years [1–4]. More severe CF transmembrane conductance regulator (CFTR) mutations are associated with poorer clinical outcomes [5]; however, identification of exogenous factors that contribute to the inflammatory process and susceptibility to infection may provide an opportunity for intervention.

Both gaseous and solid components of air pollution are associated with adverse clinical outcomes in the CF population [6–10], and fine particulate matter (PM_2.5_; atmospheric particles with an aerodynamic diameter ≤ 2.5 μm), has consistently been shown to be a predictor of pulmonary morbidities [6, 8–10]. Declining lung function and increased pulmonary exacerbation rates [8] have been reported in association with long-term exposure to PM_2.5_, and increased risk of pulmonary exacerbations has been described for higher short-term exposure [6, 8]. To date, few studies have examined the role of air pollution exposure in increasing susceptibility to respiratory pathogens in CF patients [9, 10]. We recently demonstrated that chronic PM_2.5_ exposure is associated with an increased risk of initial *Pseudomonas aeruginosa* acquisition in young children with CF [10]. The purpose of this study was to evaluate early life fine particulate matter exposure and risk of initial acquisition of other CF pathogens, including methicillin-susceptible *Staphylococcus aureus* (MSSA), methicillin-resistant *Staphylococcus aureus* (MRSA), *Stenotrophomonas maltophilia,* and *Achromobacter xylosoxidans* in the same population of young children with CF.

## Methods

### Study population and setting

We performed a retrospective cohort study to evaluate the association between early life PM_2.5_ exposure and time to initial acquisition of commonly cultured bacteria from the respiratory tract of U.S. children <6 years of age with CF, including MSSA, MRSA, *S. maltophilia*, and *A*. *xylosoxidans*, using the U.S. Cystic Fibrosis Foundation National Patient Registry, 2003–2009. The registry contains detailed encounter-based information on individual-level demographic and disease characteristics for all patients treated at CF Foundation-accredited care centers [11].

The study population consisted of all children residing in the lower 48 states born after December 31, 2002 and with a first encounter and respiratory culture recorded prior to 2 years of age. To evaluate incident pathogen acquisition, those children whose initial culture was positive for the pathogen of interest were excluded from that analysis. This study was approved by the Institutional Review Board of the University of Washington and the Cystic Fibrosis Foundation Registry Committee.

### Particulate matter exposure

The primary exposure of interest was the mean annual concentration of PM_2.5_ in the calendar year prior to birth for each child. Year prior to birth was chosen because it captured PM_2.5_ exposure prior to disease onset and accounted for the known secular decline in U.S. air pollution levels that occurred during this time period.

Annual summary measures of PM_2.5_ were obtained from the U.S. Environmental Protection Agency Air Quality System (https://www.epa.gov/aqs), a national network of federally and locally funded air monitoring sites located throughout the U.S. Over the course of the study, 1574 monitoring stations came on-line and went off-line; only data from those monitors in operation for an entire year with no more than a 45-day gap between measurements, which ranged from 73% of monitors in 2003 to 81% in 2002 and 2007, were utilized for analyses.

Individual-level PM_2.5_ exposure for each child was assigned in the following manner. Initially, latitude and longitude coordinates for each of the monitoring stations were geocoded using ArcGIS 10.1 (ESRI, Redlands, CA). A similar procedure was then used to geocode each child’s residence, taken as the centroid of the residential zip code at the time of entry into the registry. Individual-level PM_2.5_ exposure was assigned using 4 common PM_2.5_ exposure metrics. First, PM_2.5_ exposure was determined based on a nearest monitor approach in which the closest monitoring station (based on linear distance) was identified for each of the residential zip code centroids; the corresponding PM_2.5_ concentration of the monitor served as the individual-level exposure. Inverse distance weighting (IDW) procedures were then employed to estimate three PM_2.5_ exposures. For the IDW approach, exposure was estimated as the weighted value (based on an exponential decay model over distance) of PM_2.5_ concentrations of monitors located within 50, 30, and 10 miles of the residential zip code centroid. Individuals for whom no monitoring station was located within each of these distances were excluded from analyses. The primary PM_2.5_ exposure metric was based on the IDW within 30 miles, in accordance with the previously published CF literature [8–10]; however, complete results for each metric are provided.

### Outcomes

The primary outcomes were time to first positive culture for MSSA, MRSA, *S*. *maltophilia*, and *A*. *xylosoxidans* recorded in the registry. CF Foundation guidelines of care recommend quarterly (i.e., four times annually) respiratory cultures [12], typically obtained from oropharyngeal swabs in this young, non-expectorating population. Because the exact date of acquisition is not known with this surveillance approach, pathogen acquisition was defined as occurring in the interval between the date of previous negative culture (left hand endpoint) and the date of first positive respiratory culture (right hand endpoint).

### Statistical analysis

Demographic and clinical characteristics were compared between children acquiring each pathogen and those that remained pathogen-free during follow-up using Student *t* tests with unequal variances for continuous variables and Chi square tests for categorical variables.

Multivariable Weibull regression with interval censored outcomes [13] was used to evaluate the association between mean annual PM_2.5_ concentration in the year prior to birth and time to first recorded positive culture for each pathogen. For each analysis, children entered the study upon first clinical encounter recorded in the registry. Subjects were right censored on the date of the last encounter prior to December 31, 2009 if they remained pathogen-free during follow-up. All analyses were adjusted a priori for the following potential confounders: sex, race (white vs. non-white), ethnicity (Hispanic vs. non-Hispanic), insurance status (any private insurance vs. no private insurance), urban/rural status using the Rural Urban Commuting Area coding Version 2.0 [14] (defined as urban, large rural, small rural, or isolated), age at diagnosis of CF, diagnosis by newborn screening, and CFTR functional class, defined as follows: Severe, both CFTR mutations result in minimal CFTR function (class 1, 2, or 3), including F508 del; Residual, at least one allele with a mutation resulting in partial CFTR function (class 4 or 5); Unclassified, both alleles with unknown functional class, or one allele with minimal CFTR function and the second with unknown functional class. Results of multivariable models are presented as hazard ratios (HR) and corresponding 95% confidence intervals (CI) for each 10 μg/m^3^ increase in PM_2.5_ exposure. A two-sided *P* value <0.05 was considered statistically significant. All analyses were performed using R (Version 3.0.2) [15].

## Results

**Fig. 1:**
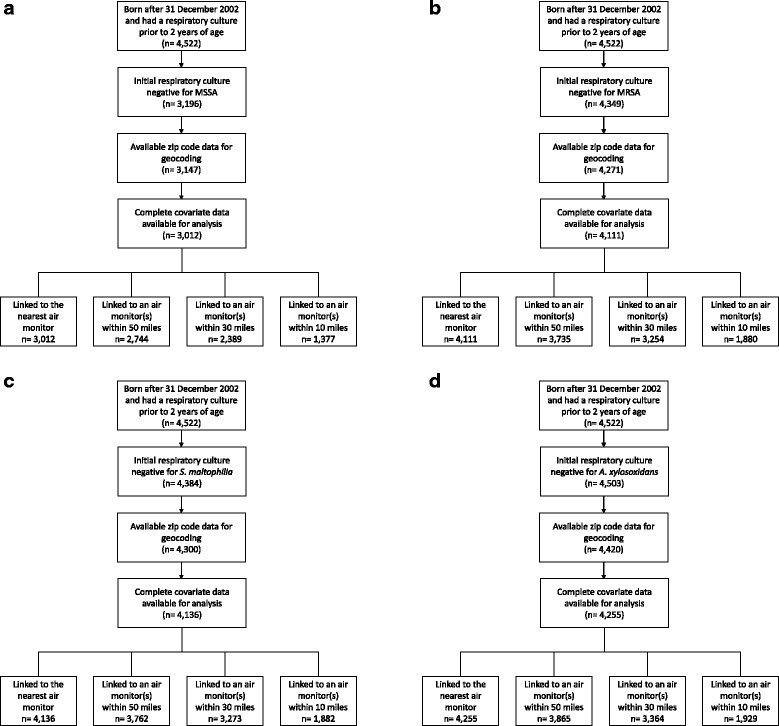
Flow chart of study cohorts for evaluation of the association of PM_2.5_ exposure and acquisition of **a**) methicillin susceptible *Staphylococcus aureus*, **b**) methicillin resistant *Staphylococcus aureus*, **c**) *Stenotrophomonas maltophilia,* and **d**) *Achromobacter xylosoxidans*

A total of 4522 children in the CFF Registry were born after December 31, 2002 and had a first registry encounter and respiratory culture recorded prior to 2 years of age. Of these 3463 (77%) had PM_2.5_ data from the year prior to birth available from a monitoring station within 30 miles of the residence location and comprised the study population. The cohorts for each of the pathogens investigated varied slightly due to the inclusion of only incident cases (Fig. 1). A total of 1885 of 3012 (63%) children acquired MSSA, 706 out of 4111 (17%) acquired MRSA; 992 of 4136 (24%) acquired *S*. *maltophilia*; and 201 of 4255 (5%) acquired *A*. *xyloxidans* while under study. The median time to initial acquisition amongst those who acquired these pathogens was 20 months (25th–75th percentiles: 13, 32 months), 30 months (25th–75th percentiles: 17, 46 months), 23 months (15, 37 months), and 34 months (20, 49 months), respectively.

Table 1 compares the demographic and clinical characteristics of those children acquiring and remaining free of each organism during follow-up. Children acquiring each pathogen were more likely to have severe CFTR mutations and less likely to be diagnosed by newborn screening than those remaining pathogen-free. Children with any private insurance were less likely to acquire MRSA, *S*. *maltophilia*, and *A. xylosoxidans* compared to children with no private insurance. Males were more likely than females to acquire MSSA and *A. xylosoxidans*, while a greater proportion of Hispanic children acquired MRSA and *A. xylosoxidans* than did non-Hispanics.

**Table 1:** Demographic and clinical characteristics of young children with cystic fibrosis by pathogen acquisition status, 2003–2009

	Respiratory Pathogen											
	MSSA			MRSA			*S. maltophilia*			*A*. *xylosoxidans*		
	Acquired (*n* = 1885)	Negative (*n* = 1127)	*P* value	Acquired (*n* = 706)	Negative (*n* = 3405)	*P* value	Acquired (*n* = 992)	Negative (*n* = 3144)	*P* value	Acquired (*n* = 201)	Negative (*n* = 4054)	*P* value
Male (%)	909 (48)	589 (52)	0.04	347 (49)	1731 (51)	0.44	502 (51)	1581 (50)	0.89	86 (43)	2061 (51)	0.03
White (%)	1750 (93)	1019 (90)	0.02	636 (90)	3134 (92)	0.10	913 (92)	2887 (92)	0.88	179 (89)	3726 (92)	0.19
Hispanic (%)	201 (11)	121 (11)	0.99	50 (7)	403 (12)	<0.01	124 (13)	327 (10)	0.07	35 (17)	430 (11)	<0.01
Any Private Insurance (%)	984 (52)	591 (52)	0.93	301 (43)	1903 (56)	<0.01	471 (47)	1721 (55)	<0.01	79 (39)	2174 (54)	<0.01
Age at diagnosis, months, mean (SD)	2.5 (4.3)	2.2 (4.3)	0.12	2.7 (4.3)	2.3 (4.2)	0.66	2.5 (4.2)	2.4 (4.3)	0.26	3.2 (4.8)	2.4 (4.2)	0.03
Identified by newborn screening (%)	663 (35)	611 (54)	<0.01	213 (30)	1555 (46)	<0.01	358 (36)	1414 (45)	<0.01	68 (34)	1756 (43)	<0.01
CFTR functional class^a^ (%)												
Severe	1275 (68)	714 (63)	0.04	514 (73)	2148 (63)	<0.01	695 (70)	1991 (63)	<0.01	134 (67)	2638 (65)	0.03
Residual	156 (8)	116 (10)		45 (6)	328 (10)		60 (6)	315 (10)		8 (4)	373 (9)	
Unclassified	454 (24)	297 (26)		147 (21)	929 (27)		237 (24)	838 (27)		59 (29)	1043 (26)	

Data given as No. (%) unless otherwise indicated, *MSSA* methicillin susceptible *Staphylococcus aureus*, *MRSA* methicillin resistant *Staphylococcus aureus*, *S maltophilia*, *Stenotrophomonas maltophilia*, *A*. *xylosoxidans*, *Achromobacter xylosoxidans*

^a^ CFTR Mutation class is defined as follows: Severe, includes children in which both CFTR allele mutations result in minimal CFTR function (class 1, 2, or 3), including F508 del; Residual, children for which at least one allele with a mutation resulting in partial CFTR function is present (class 4 or 5); Unclassified, both alleles with unknown functional class, or one allele with minimal CFTR function and the second with unknown functional class

Mean annual PM_2.5_ concentrations for the study population in the year prior to birth by acquisition status of each pathogen are presented in Table 2. For all metrics of PM_2.5_ exposure and for each pathogen, children who had a positive culture during follow-up had a higher mean PM_2.5_ exposure in the year prior to birth compared to those that remained pathogen-free.

**Table 2:** Mean and standard deviations of PM_2.5_ concentrations (μg/m^3^) in year prior to birth for young children with cystic fibrosis from 2003 to 2009, overall and by methicillin susceptible *Staphylococcus aureus*, methicillin resistant *Staphylococcus aureus*, *Stenotrophomonas maltophilia,* and *Achromobacter xylosoxidans* acquisition status

	Respiratory Pathogen											
	MSSA			MRSA			*S. maltophilia*			*A. xylosoxidans*		
	Overall	Acquired	Negative	Overall	Acquired	Negative	Overall	Acquired	Negative	Overall	Acquired	Negative
*PM2.5 Metric*												
Nearest monitor^a^	12.04 (2.75)	12.20 (2.77)	11.77 (2.71)	12.06 (2.79)	12.55 (2.62)	11.95 (2.81)	12.06 (2.78)	12.46 (2.67)	11.94 (2.81)	12.06 (2.78)	12.65 (2.48)	12.03 (2.79)
IDW: 50 miles^b^	12.27 (2.58)	12.45 (2.60)	11.99 (2.52)	12.27 (2.60)	12.83 (2.39)	12.15 (2.63)	12.27 (2.60)	12.65 (2.48)	12.16 (2.63)	12.28 (2.60)	12.86 (2.30)	12.25 (2.61)
IDW: 30 miles^c^	12.29 (2.66)	12.46 (2.68)	12.01 (2.59)	12.31 (2.68)	12.85 (2.50)	12.21 (2.70)	12.31 (2.68)	12.69 (2.55)	12.19 (2.71)	12.32 (2.67)	12.95 (2.40)	12.29 (2.40)
IDW: 10 miles^d^	12.26 (2.76)	12.40 (2.79)	12.04 (2.69)	12.31 (2.81)	12.96 (2.73)	12.19 (2.80)	12.30 (2.80)	12.64 (2.71)	12.20 (2.82)	12.32 (2.80)	13.08 (2.50)	12.29 (2.81)

*MSSA* methicillin susceptible *Staphylococcus aureus*, *MRSA* methicillin resistant *Staphylococcus aureus*, *S maltophilia*, *Stenotrophomonas maltophilia*, *A xylosoxidans*, *Achromobacter xylosoxidans*, *PM*_*2.5*_ particulate matter ≤2.5 μm in aerodynamic diameter, *IDW* inverse distance weighted

^a^ Subjects with available PM_2.5_ data (nearest monitor), by respiratory pathogen: MSSA (*n* = 3012), MRSA (*n* = 4111), *S*. *maltophilia* (*n* = 4136) and *A*. *xylosoxidans* (*n* = 4255)

^b^ Subjects with available PM_2.5_ data (IDW: 50 miles), by respiratory pathogen: MSSA (*n* = 2744), MRSA (*n* = 3735), *S*. *maltophilia* (*n* = 3762) and *A*. *xylosoxidans* (*n* = 3865)

^c^ Subjects with available PM_2.5_ data (IDW: 30 miles), by respiratory pathogen: MSSA (*n* = 2389), MRSA (*n* = 3254), *S*. *maltophilia* (*n* = 3273) and *A*. *xylosoxidans* (*n* = 3364)

^d^ Subjects with available PM_2.5_ data (IDW: 10 miles), by respiratory pathogen: MSSA (*n* = 1377), MRSA (*n* = 1880), *S*. *maltophilia* (*n* = 1882) and *A*. *xylosoxidans* (*n* = 1929)

Results of the Weibull regression evaluating the association of PM_2.5_ exposure and risk of acquisition of respiratory pathogens are presented in Table 3. For each 10 μg/m^3^ increase in PM_2.5_ exposure, there was a statistically significantly increased risk of MRSA acquisition (HR = 1.56; 95% CI: 1:13, 2.14). PM_2.5_ exposure was not associated with increased risk of MSSA (HR = 0.97; 95% CI: 0.80, 1.17), *S*. *maltophilia* (HR = 1.28; 95% CI: 0.99, 1.66) or *A. xylosoxidans* (HR = 1.42; 95% CI: 0.78, 2.58) acquisition. Similar results were obtained in the analysis of the nearest monitor and IDW 50- and 10-mile PM_2.5_ metrics; however, in the nearest monitor PM_2.5_ exposure analysis, PM_2.5_ was associated with an increased risk of *S*. *maltophilia* acquisition (HR = 1.34; 95% CI: 1.07, 1.68).

**Table 3:** Results of multivariable Weibull regression with interval censored outcomes^1^ evaluating the association of PM_2.5_ exposure and time to methicillin susceptible *Staphylococcus aureus*, methicillin resistant *Staphylococcus aureus*, *Stenotrophomonas maltophilia,* and *Achromobacter xylosoxidans* acquisition for young children with cystic fibrosis, 2003–2009

PM_2.5_ metric				
	Nearest monitor	IDW: 50 miles	IDW: 30 miles	IDW: 10 miles
	HR (95% CI)	HR (95% CI)	HR (95% CI)	HR (95% CI)
MSSA	0.91 (0.77, 1.08)	0.93 (0.77, 1.13)	0.97 (0.80, 1.17)	0.90 (0.71, 1.15)
MRSA	*1.48 (1.14, 1.93)*	*1.68 (1.24, 2.27)*	*1.56 (1.13, 2.14)*	*1.78 (1.17, 2.69)*
*S*. *maltophilia*	*1.34 (1.07, 1.68)*	1.30 (1.00, 1.65)	1.28 (0.99, 1.66)	1.10 (0.79, 1.53)
*A*. *xylosoxidans*	1.48 (0.91, 2.41)	1.44 (0.83, 2.53)	1.42 (0.78, 2.58)	1.51 (0.68, 3.34)

*PM*_*2.5*_ particulate matter ≤2.5 μm in aerodynamic diameter, *IDW* inverse distance weighted, *HR* hazard ratio, *CI* confidence interval, *MSSA* methicillin susceptible *Staphylococcus aureus*, *MRSA* methicillin resistant *Staphylococcus aureus*, *S maltophilia Stenotrophomonas maltophilia*, *A xylosoxidans*, *Achromobacter xylosoxidans*

^1^All regression models were adjusted for: sex, race, ethnicity, insurance status, rural urban commuting area, diagnosis by newborn screening, age at diagnosis of CF, and CFTR mutation class. Results of regression models reflect the hazard ratio associated with a 10 μg/m^3^ increase in PM_2.5_ exposure. Hazard ratios that are significantly different from 1.00 (*P* < 0.05) are in italics

## Discussion

In this large, national cohort of U.S. CF patients <6 years of age, PM_2.5_ exposure was associated with an increased risk of initial acquisition of MRSA (but not of the other organisms we examined), with a 56% (95% CI: 13%, 114%) increased risk of MRSA acquisition for each 10 μg/m^3^ increase in PM_2.5_. We recently reported a similar association between PM_2.5_ exposure and risk of initial *P. aeruginosa* acquisition in the same cohort, with a 24% (95% CI: 1–51%) increased risk of *P. aeruginosa* acquisition for each 10 μg/m^3^ increase of PM_2.5_ in the year prior to birth, after adjustment for covariates [10]. Similarly, Collaco et al. [9] reported increased odds of *P. aeruginosa* prevalence (Odds Ratio [OR] for each 10 μg/m^3^ increase in PM_2.5_ = 1.12; 95% CI: 1.01, 1.23) among 677 patients in the U.S. CF Registry, employing the average PM_2.5_ exposure in 2006 based on the nearest monitor within 30 miles of residential zip code. Our findings suggest that air pollution is an independent risk factor for and may play a previously unrecognized role in respiratory colonization by MRSA and *P. aeruginosa* in the CF population.

Chronic infection with MRSA is associated with poorer clinical outcomes and survival in CF patients [16–18]. Of concern, MRSA prevalence among U.S. CF patients has increased steadily from 9% in 2002 to 27% in 2012 [19]. About 70% of MRSA isolates among children with CF in the US are “health care–associated” (SCCmec II) versus “community-associated” (SCCmec IV) strains [20], though the prevalence of SCCmecIV relative to SCCmecII strains has increased over the last decade. Known risk factors for MRSA acquisition in CF patients include colonization with *P. aeruginosa* [20], more frequent clinic visits [20] and higher mean ambient temperature [21]. To our knowledge, the association of air pollution exposure and risk of MRSA acquisition has not previously been evaluated.

The mechanism by which exposure to PM_2.5_ may increase the risk of MRSA acquisition among young CF patients deserves further exploration. As there are no data to suggest that components of air pollution directly increase exposure to MRSA or other pathogens, it seems more likely that adverse effects of PM_2.5_ exposure on the CF airway increases susceptibility to MRSA infection. Limited in vitro studies exist regarding the biologic mechanisms by which exposure to fine particulate matter adversely affects the CF airway. Kamdar and colleagues demonstrated that PM_2.5_ increases oxidative stress and mitochondrial signaling-mediated apoptosis in CF human bronchial epithelial cells [22] which in turn could increase airway inflammation. Further, Geiser et al. demonstrated higher uptake of inhaled nanoparticles by alveolar epithelial cells and increased inflammatory response of CFTR mutant mice compared to wild type [23]. The effects of gaseous air pollutants on the CF airway are less well understood; however, increased ozone levels have been shown to downregulate CFTR function in human bronchial epithelial cells [24].

Similarly, epidemiological studies have demonstrated adverse clinical outcomes associated with short and long-term air pollution exposure among CF patients, as well as for specific constituents of air pollution. In the first such investigation, utilizing the U.S. CF Registry, Goss, et al. [8] reported increased pulmonary exacerbations associated with the long term exposure to PM_10_, PM_2.5_ and ozone, with declining lung function and FEV_1_ associated with increased PM_2.5_ exposure. Subsequently, Goeminne, et al. [6] conducted a case-crossover study in a cohort of Belgium CF patients and reported that short-term exposure to PM_10_ (OR = 1.04; 95% CI: 1.00–1.08), ozone (OR = 1.11; 95% CI: 1.05–1.17) and NO_2_ (OR = 1.03; 95% CI: 1.00–1.07) were associated with increased risk of pulmonary exacerbations. Farhat, et al. reported an increased risk of pulmonary exacerbation with a two-day lagged ozone exposure (Relative Risk = 1.86; 95% CI, 1.14–3.02) in a longitudinal analysis of Brazilian CF patients in a metropolitan city [7]. However, Jassel, et al. reported no association of PM_2.5_, ozone or proximity to major road ways and frequency of pulmonary exacerbations in a cohort of 145 children with CF [25].

In the general population, prenatal exposure to air pollution is a risk factor for low birth weight [26, 27], which in turn is a risk factor for adverse respiratory outcomes in early childhood [28]. In addition, post-natal air pollution exposure has been associated with an increased risk of bronchiolitis and recurrent wheeze in infants and young children [29, 30]. Our findings of increased risk of MRSA acquisition in CF patients may be in part mediated by these same risk factors, particularly since PM_2.5_ exposure was measured in the year prior to birth.

Environmental factors are associated with approximately 50% of the population variability in lung function [31] and *P. aeruginosa* acquisition [32] in CF patients. Many of the CF-related pathogens are naturally occurring in the environment, although few studies have investigated specific environmental factors that may contribute to pathogen acquisition [33]. *P. aeruginosa* is the most widely studied CF-related bacterium with seasonal variations in acquisition [34] and differential geographic residual relative risk [13] reported. These same factors may contribute to MRSA acquisition in CF patients.

Strengths of the present investigation include the large, national cohort with regular respiratory culture results. There are also several limitations. First, available PM_2.5_ data was limited to outdoor exposure. Therefore, evaluation of cumulative exposure to PM_2.5_ including indoor air pollution was not possible. Similarly, the gaseous components of air pollution (e.g. ozone and nitrous oxides) as well as coarse particulate matter (PM_2.5–10_), were not considered. Second, PM_2.5_ exposure was based upon a nearest monitor approach rather than monitoring at the patient’s residence, which may have resulted in misclassification of PM_2.5_ exposure. Furthermore, as air pollution exposure was determined in the calendar year prior to birth, change in zip code of residence during follow-up may not accurately represent long-term PM_2.5_ exposure. Third, information on environmental tobacco smoke exposure, preterm birth/low birth weight, or maternal occupational exposures, potentially important variables, were not available. Fourth, information on MRSA strains (SCCmec type or Panton Valentine Leukocidin status) was unavailable so we were not able to distinguish community vs. healthcare associated strains. It is possible that the risk of MRSA acquisition associated with PM_2.5_ exposure may differ by subtypes. Finally, respiratory cultures were performed primarily on oropharyngeal swabs in the non-expectorating young cohort. The specificity and, even more, the sensitivity of oropharyngeal cultures in comparison to lower respiratory samples is limited [35]; results of this study may not be generalizable to lower airway colonization. Nonetheless, oropharyngeal swabs are standard of care for assessment of respiratory cultures in pre-expectorating patients in the U.S., and acquisition of pathogens in the upper airway is generally considered an important clinical outcome.

## Conclusions

In conclusion, increasing levels of exposure to fine particulate matter was found to be a risk factor for initial MRSA acquisition in young children with CF. These results further strengthen the growing evidence that increased levels of air pollution are associated with adverse outcomes in the CF population. Additional studies that investigate the impact of air pollution on other CF-related outcomes in young children are recommended. Given the morbidity associated with CF chronic respiratory infections and lack of strategies available to patients to prevent them, future studies that can elucidate other risk factors for these infections are needed.

## Abbreviations

*A. xylosoxidans*: *Achromobacter xylosoxidans*
CF: cystic fibrosis
CFTR: cystic fibrosis transmembrane conductance regulator
CI: confidence interval
FEV_1_: forced expiratory volume in one second
HR: hazard ratio
IDW: inverse distance weighted
MRSA: methicillin-resistant *Staphylococcus aureus*
MSSA: methicillin-susceptible *Staphylococcus aureus*
NO_2_: nitrogen dioxide
OR: odds ratio
*P. aeruginosa*: *Pseudomonas aeruginosa*
PM_10_: particulate matter ≤10 μm in aerodynamic diameter
PM_2.5_: particulate matter ≤2.5 μm in aerodynamic diameter
*S. maltophilia*: *Stenotrophomonas maltophilia*
SD: standard deviation
